# *FAR1* and *FAR2* Regulate the Expression of Genes Associated with Lipid Metabolism in the Rice Blast Fungus *Magnaporthe oryzae*

**DOI:** 10.1371/journal.pone.0099760

**Published:** 2014-06-20

**Authors:** Mohammad Termizi bin Yusof, Michael J. Kershaw, Darren M. Soanes, Nicholas J. Talbot

**Affiliations:** 1 School of Biosciences, University of Exeter, Exeter, United Kingdom; 2 Department of Microbiology, Faculty of Biotechnology and Biomolecular Sciences, Universiti Putra Malaysia, UPM Serdang, Serdang, Selangor, Malaysia; Nanjing Agricultural University, China

## Abstract

The rice blast fungus *Magnaporthe oryzae* causes plant disease via specialised infection structures called appressoria. These dome-shaped cells are able to generate enormous internal pressure, which enables penetration of rice tissue by invasive hyphae. Previous studies have shown that mobilisation of lipid bodies and subsequent lipid metabolism are essential pre-requisites for successful appressorium-mediated plant infection, which requires autophagic recycling of the contents of germinated spores and germ tubes to the developing appressorium. Here, we set out to identify putative regulators of lipid metabolism in the rice blast fungus. We report the identification of *FAR1* and *FAR2*, which encode highly conserved members of the Zn2-Cys6 family of transcriptional regulators. We generated *Δfar1*, *Δfar2* and *Δfar1Δfar2* double mutants in *M. oryzae* and show that these deletion mutants are deficient in growth on long chain fatty acids. In addition, *Δfar2* mutants are also unable to grow on acetate and short chain fatty acids. *FAR1* and *FAR2* are necessary for differential expression of genes involved in fatty acid β-oxidation, acetyl-CoA translocation, peroxisomal biogenesis, and the glyoxylate cycle in response to the presence of lipids. Furthermore, *FAR2* is necessary for expression of genes associated with acetyl-CoA synthesis. Interestingly, *Δfar1*, *Δfar2* and *Δfar1Δfar2* mutants show no observable delay or reduction in lipid body mobilisation during plant infection, suggesting that these transcriptional regulators control lipid substrate utilization by the fungus but not the mobilisation of intracellular lipid reserves during infection-related morphogenesis.

## Introduction

Rice blast disease is caused by the fungus *Magnaporthe oryzae* and is one of the most destructive diseases of cultivated rice. Serious harvest losses can occur in all rice-growing regions of the world, with up to 18% yield losses per annum [1]. The prevention of rice blast epidemics is therefore key to maintaining and improving rice production to ensure global food security. The spread of rice blast disease occurs by dispersal of asexual spores, called conidia, which adhere strongly to the leaf surface. A conidium germinates on the leaf cuticle and develops a polarised germ tube, which swells at its tip to form a single-celled appressorium. The appressorium accumulates high concentrations of osmotically compatible solutes, including glycerol, which generates huge internal pressure that the fungus utilises to force a narrow penetration hypha through the plant cuticle and into the leaf epidermis [2].

Appressorium formation occurs in water on the surface of the rice leaf, where there are no available nutrients. Therefore, to synthesise the high concentrations of glycerol necessary for plant infection, the fungus needs to mobilise energy storage reserves from the conidium [1]. Trehalose, glycogen and lipids are among the major storage compounds in spores and previous studies have implicated glycogen and lipids as precursors for glycerol synthesis in appressoria of *M. oryzae* [3], [4]. Trehalose synthesis and glycogen metabolism have been shown to be required for fungal virulence [5] [6]. Trehalose-6-phosphate synthase, for example, is a key regulator of virulence in *M. oryzae*, that controls glucose-6-phosphate levels and the balance of NADPH/NADP in fungal cells during plant infection [7].

Lipid bodies are abundant in germinating spores of *M. oryzae* and move to the apex of the germ tube in a process regulated by the Pmk1 MAPK signalling pathway [4]. Lipid bodies then coalesce before being taken up by vacuoles within the maturing appressorium [8]. At the onset of turgor generation in the appressorium, lipid bodies then undergo rapid degradation in an autophagic process, regulated at least in part by the cAMP-dependent PKA pathway [4]. Ultimately, the entire contents of the conidium and germ tube are degraded by macroautophagy, resulting in collapse and death of the spore and transfer of its contents to the incipient appressorium [9], [10]. Plant infection-associated autophagy is necessary for establishment of rice blast disease [9], [10].

Lipid breakdown is catalyzed by triacylglycerol lipases, yielding both glycerol and fatty acid. Triacylglycerol lipase activity is highly induced in *M. oryzae* during the onset of appressorium formation, remaining high throughout turgor generation [4]. The fungus possesses a total of 19 genes encoding intracellular lipases, but targeted deletion of any one of these genes individually does not affect plant infection by *M. oryzae*, suggesting that lipid breakdown involves the coordinated action of more than one lipase [11]. The liberation of fatty acids during spore germination also suggests a significant requirement for fatty acid β-oxidation during infection-related development, where fatty acids are activated to the corresponding acyl coenzyme A (CoA) and then oxidised via a four-step pathway. Genomic analysis in *M. oryzae* identified a set of genes predicted to encode enzymes involved in fatty acid β-oxidation [11] and showed that deletion of the peroxisome-localised *MFP1*, which encodes the multifunctional β-oxidation protein, is necessary for appressorium function and virulence [11], along with components of the peroxisomal biogenesis pathway and carnitine acetyl transferase [12], [13]. Fatty acid β-oxidation results in generation of acetyl-CoA, which must be converted via the glyoxylate bypass to allow gluconeogenesis and cell wall biosynthesis, for instance. The glyoxylate cycle is important during infection by *M. oryzae*. Mutants lacking *ICL1*, which encodes isocitrate lyase, show a delay in generation of symptoms of rice blast disease [14]. This is consistent with studies in a range of fungal and bacterial pathogens which have shown that the glyoxylate cycle is a key determinant of successful disease establishment in a wide variety of pathogens [15] [16] [18]. Recent evidence in *M. oryzae* also underlines a significant role for the glyoxylate cycle based on analysis of the peroxisomal alanine-glyoxylate aminotransferase, which is necessary for lipid mobilization and utilization during appressorium development [17].

In this study, we set out to identify potential regulators of lipid metabolism in *M. oryzae* and to determine their role in the fungus. In *Aspergillus nidulans*, two genes, FarA and FarB, have been identified and shown to regulate induction of genes involved in fatty acid β-oxidation, the glyoxylate cycle and peroxisomal biogenesis [18]. Significantly, *Fusarium oxysporum* and *Candida albicans* also possess orthologues of the Far proteins involved in regulating lipid metabolism [19], [20]. In this report, we describe the identification of two putative regulators of lipid metabolism and peroxisome function in *M. oryzae, FAR1* and *FAR2*. We have carried out targeted deletion of these genes and also generated a mutant lacking both putative regulators. We show that the ability to utilize fatty acids as a carbon source is dependent on the presence of *FAR1* and *FAR2.* We also demonstrate that *FAR1 and FAR2* are necessary for the differential expression of genes involved in fatty acid β-oxidation, acetyl-CoA translocation, peroxisome biogenesis, and the glyoxylate cycle in response to the presence of lipids.

## Results

### *FAR1* and *FAR2* Encode Putative Transcriptional Regulators

**Figure 1:**
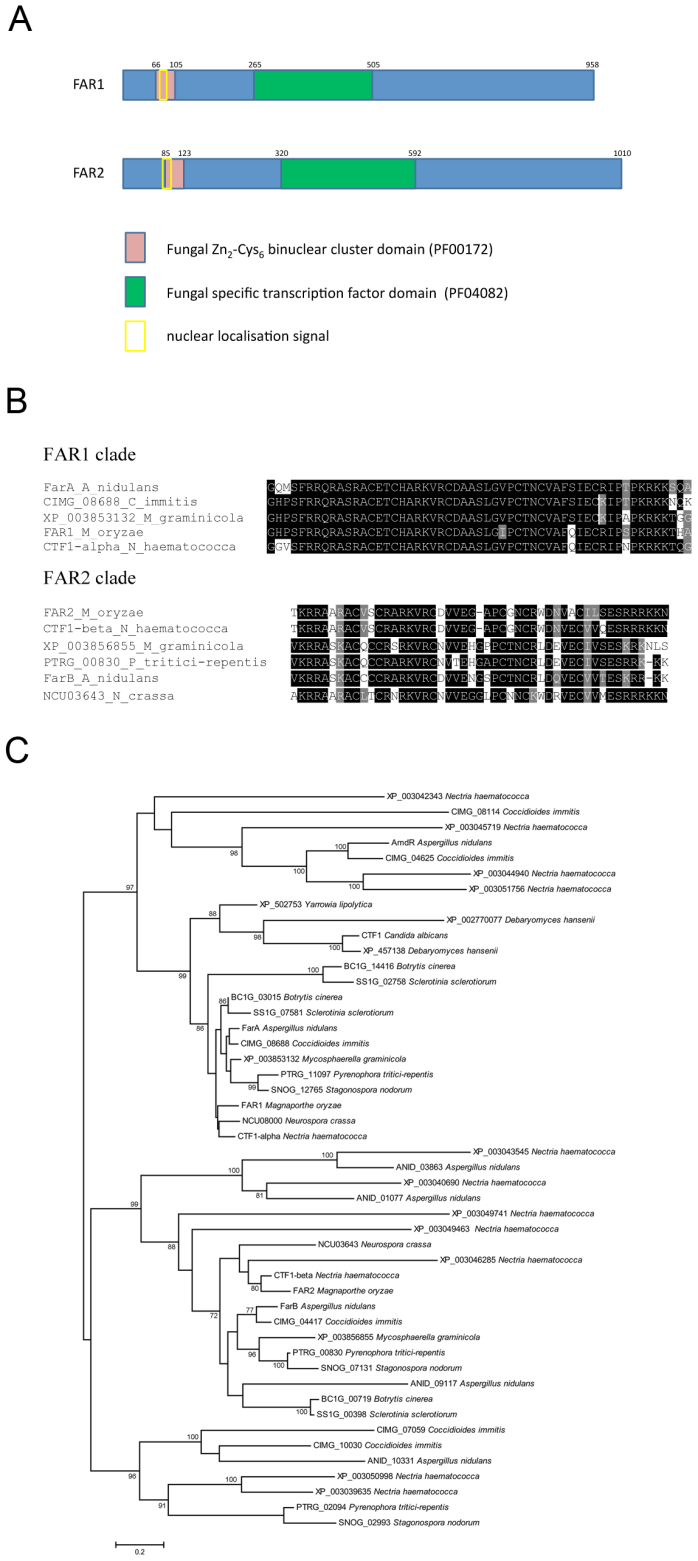
Characterization of Far1 and Far2 from *M. oryzae*. **A.** Schematic diagram to show domains found in protein sequences of Far1 and Far2 identified using Pfam (accession numbers shown in brackets in key) and Prosite scans. Amino acid positions are marked on the diagram. **B**. Multiple sequence alignment (using clustal) showing Zn_2_-Cys_6_ binuclear cluster domain in selected sequences from *FAR1* and *FAR2* clade. **C**. Maximum likelihood phylogenetic tree created using PhyML. Bootstrap support values of 70 or greater are indicated on tree.

Bioinformatic analysis revealed two genes, *FAR1* (MGG_01836.6) and *FAR2* (MGG_08199.6) in the *Magnaporthe* genome database (http://www.broadinstitute.org/), that are putative orthologues of the *A. nidulans* FarA and FarB transcriptional regulators. The predicted *FAR1* and *FAR2* gene products possess two domains; a fungal Zn_2-_Cys_6_ binuclear cluster domain (*FAR1*, amino acids 66–106; *FAR2*, amino acids 85–124) and a fungal transcription factor domain (*FAR1*, amino acids 265–509; *FAR2*, amino acids 320–594), as shown in Figure 1A and B. The predicted *FAR1* protein has 941 amino acids and shows 58% identity to FarA, as shown in Figure S1, while the predicted *FAR2* protein has 1009 amino acids and shows 42% identity to FarB, as shown in Figure S2. A phylogenetic tree [22], Figure 1C, revealed the presence of clear *FAR1* and *FAR2* orthologues in all filamentous ascomycetes selected. Among yeast species, orthologous Far proteins were predicted in *Candida albicans*, *Debaryomyces hansenii* and *Yarrowia lipolytica*, but not in *Ashbya gossypii*, *Kluyveromyces lactis*, *Saccharomyces cerevisiae* or *Schizosaccharomyces pombe*. Furthermore, there were no homologous regulatory proteins in basidiomycetes, zygomycete or chytrid species. The tree shows a *FAR1* clade that includes FarA from *Aspergillus nidulans* [18] and CTF1-alpha from *Nectria haematococca* [21] and a *FAR2* clade that includes FarB from *Aspergillus nidulans* [18] and CTF1-beta from *Nectria haematococca* [21]. AmdR from *Aspergillus nidulans* which regulates the expression of genes involved in acetamide, omega amino acid and lactam catabolism [22] and has orthologues only in *Coccidioides immitis* and *Nectria haematococca*, is also closely related to *FAR1*.

**Figure 2:**
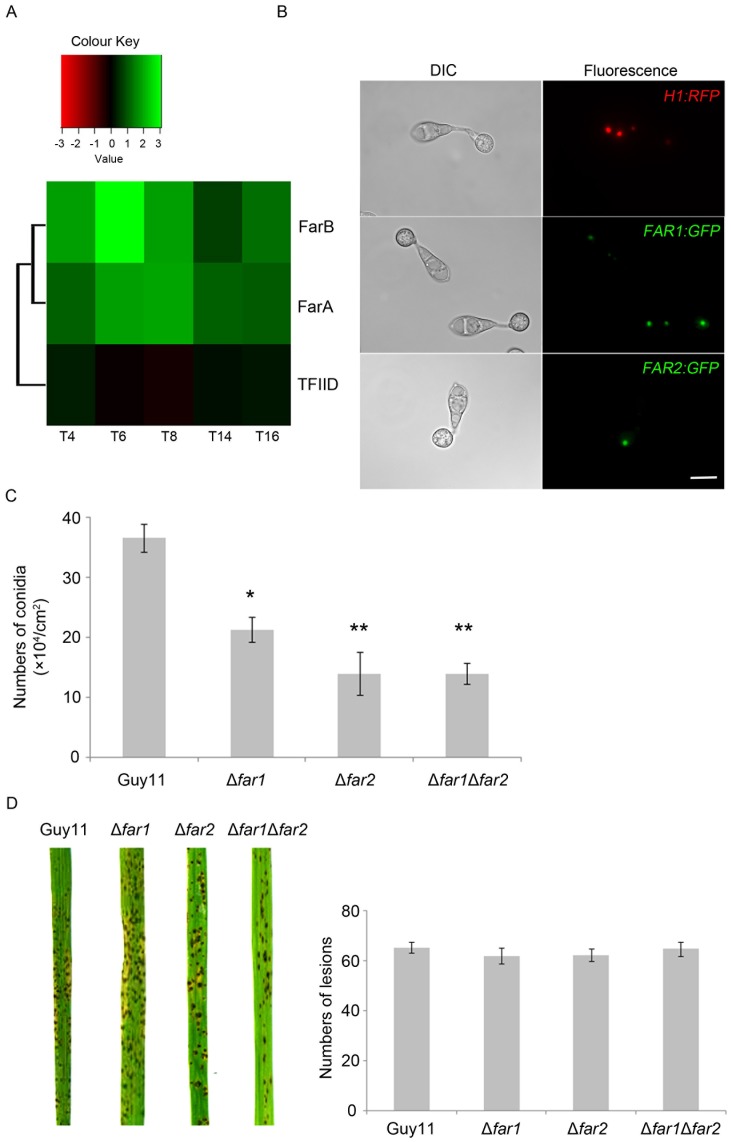
Functional characterization of *FAR1* and *FAR2* genes of *M. oryzae*. **A**. *FAR1* and *FAR2* are expressed during appressorium development in *M. oryzae*. Gene expression was evaluated using supersage analysis and a heat map is shown in which gene expression at 4h, 6h, 8h, 14h, and 16h of appressorium development is compared to expression in mycelium grown in complete media. The transcription initiation factor TFIID subunit 14 (MGG_05204) is included to indicate relative expression of a constitutively expressed gene. **B.** Epifluorescence micrographs to show nuclear localisation of FAR1-GFP, FAR2-GFP and histone H1-RFP gene fusions in *M. oryzae.* Bar = 10 µm. **C.** Bar charts to show conidiogenesis in Δ*far1*, Δ*far2* and Δ*far1*Δ*far2* mutants compared to the wild type, Guy11. Significant differences (P<0.05 or P<0.01) are indicated by one or two asterisks, respectively. **D.** Rice blast symptoms produced by Δ*far1*, Δ*far2* and Δ*far1*Δ*far2* mutants after 5 days of inoculation. Bar charts to show lesion counts from 100 infected leaves. No significant difference was observed between the mutant strains and Guy11 (P >0.01).

*FAR1* and *FAR2* were expressed throughout appressorium development (Figure 2A), showing a peak of expression between 4 to 6 h, corresponding to initial appressorium maturation. PSORT analysis (http://psort.org/) predicted that the products of *FAR1* and *FAR2* localise to the nucleus. We therefore constructed C-terminal GFP gene fusions of *FAR1* and *FAR2*, under the control of their native promoters, and expressed these in *M. oryzae*. *FAR1-GFP* and *FAR2-GFP* expression was observed throughout germination and development of the appressorium. The signal was specific to a single point in the each cell of the conidia and the appressorium, corresponding with localisation observed upon expression of histone H1-RFP (Figure 2B). Localisation of *FAR1* and *FAR2* to the nucleus is therefore consistent with their proposed role as transcriptional regulators.

### *FAR1* and *FAR2* are Necessary for Efficient Conidiogenesis in *M. oryzae*

To characterise *FAR1* and *FAR2,* we carried out targeted gene replacements using the wild type strain Guy11 as the recipient strain (Figure S3). We also generated a Δ*far1*Δ*far2* double mutant to determine the phenotypic characteristics of a mutant strain lacking both putative regulatory genes (Figure S3). The gene replacement events were analysed by Southern blot analysis and Δ*far1*, Δ*far2* and Δ*far1*Δ*far2* null mutants selected based upon the absence of hybridisation to the deleted fragment probe or a size difference of hybridising fragments as a result of the gene replacement. Targeted gene deletion mutants of Δ*far1*, Δ*far2* and Δ*far1*Δ*far2* were morphologically indistinguishable from the isogenic wild type strain Guy11. However, there was a significant (P <0.01) 40% reduction in conidiogenesis in both *Δfar1* and *Δfar2* mutants and a 60% reduction conidiation in a *Δfar1Δfar2* double mutant when compared to the wild type (Figure 2C).

To assess the virulence of *Δfar1*, *Δfar2* and the *Δfar1Δfar2* mutant, conidial suspensions of uniform concentration were sprayed onto seedlings of a susceptible rice cultivar, CO-39, and the plants were allowed to develop blast symptoms for 5 to 7 days. All mutant strains were still able to infect plants and produce necrotic lesions on the leaf surface following infection There was no delay in infection and no significant observable effects could be seen on lesion number (Figure 2D). Consistent with this, we observed that appressoria developed normally in each mutant background, developed turgor and melanin pigmentation, and were able to penetrate intact rice cuticle (data not shown). We conclude that *FAR1* and *FAR2* are necessary for efficient conidiogenesis but do not exhibit other developmental or virulence-associated phenotypes.

### *FAR1* and *FAR2* Regulate Lipid Metabolism in *M. oryzae*

**Figure 3:**
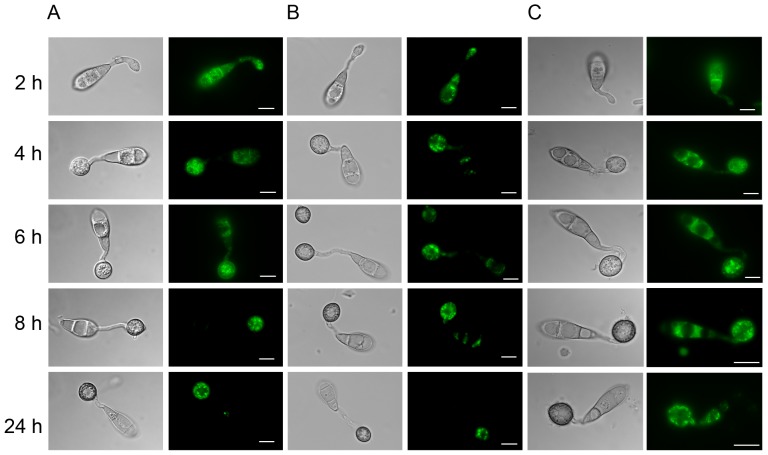
Micrographs to show lipid body mobilisation in *M. oryzae* during a time course of appressorium morphogenesis. **A.** Wild type strain Guy11, **B.** *Δfar1Δfar2* double mutant and **C.** autophagy mutant *Δatg8*. Lipid droplets in germinating conidia and appressoria were visualized by staining with Bodipy 493/503 (Invitrogen). Conidia were inoculated onto plastic cover slips in a moist chamber at 24 °C and observed for appressorium formation and lipid body mobilization at intervals, by mounting directly in fresh Bodipy 493/503 solution for 15 min. (Scale bar = 10 µm).

To investigate the role of *FAR1* and *FAR2* in lipid metabolism and lipid mobilisation we carried out a range of growth tests and cytological analyses. Lipid body mobilisation occurs during development of functional appressoria and is necessary for plant infection. In wild type strains of *M. oryzae*, lipid body mobilisation occurs as soon as the conidia adhere to the surface of a rice leaf or any hydrophobic surface. Lipid bodies were therefore visualized with BODIPY (493/503) and in conidia germinating on hydrophobic cover slips. Lipid droplets are present in conidia before germination and then accumulate at the apex of the germ tube as soon as it emerges (see Figure 3). By 4 hours, lipid bodies accumulate in the developing appressorium, and by 6 hours lipid droplets are seen in greater numbers in the appressorium compared to the conidium. By 8 hours after germination, almost all lipid droplets are present in the appressorium where lipid degradation occurs during turgor generation [4]. In Δ*far1* , Δ*far2* and Δ*far1*Δ*far2* double mutants, there was no significant difference in lipid mobilisation when compared to the wild type (Figure 3; Figure S4). We carried out more detailed quantitative analysis to determine the relative mobilisation patterns of lipid droplets in Δ*far1*, Δ*far2* and Δ*far1*Δ*far2* mutants when compared to the isogenic wild type (Figure S5), and also compared these to an autophagy-deficient *Δatg8* mutant, which showed significant delays in lipid body mobilisation and degradation (Figure 3, Figure S6), as previously reported [9] [23]. In *Δatg8* mutants the mobilisation of lipid droplets is delayed such that after 24 hours, lipid droplets are still abundant in conidia, whereas they are rapidly broken down in Guy11. We conclude that *FAR1* and *FAR2* are not necessary for infection-associated lipid body mobilisation in *M. oryzae*, which is instead dependent on autophagy.

**Figure 4:**
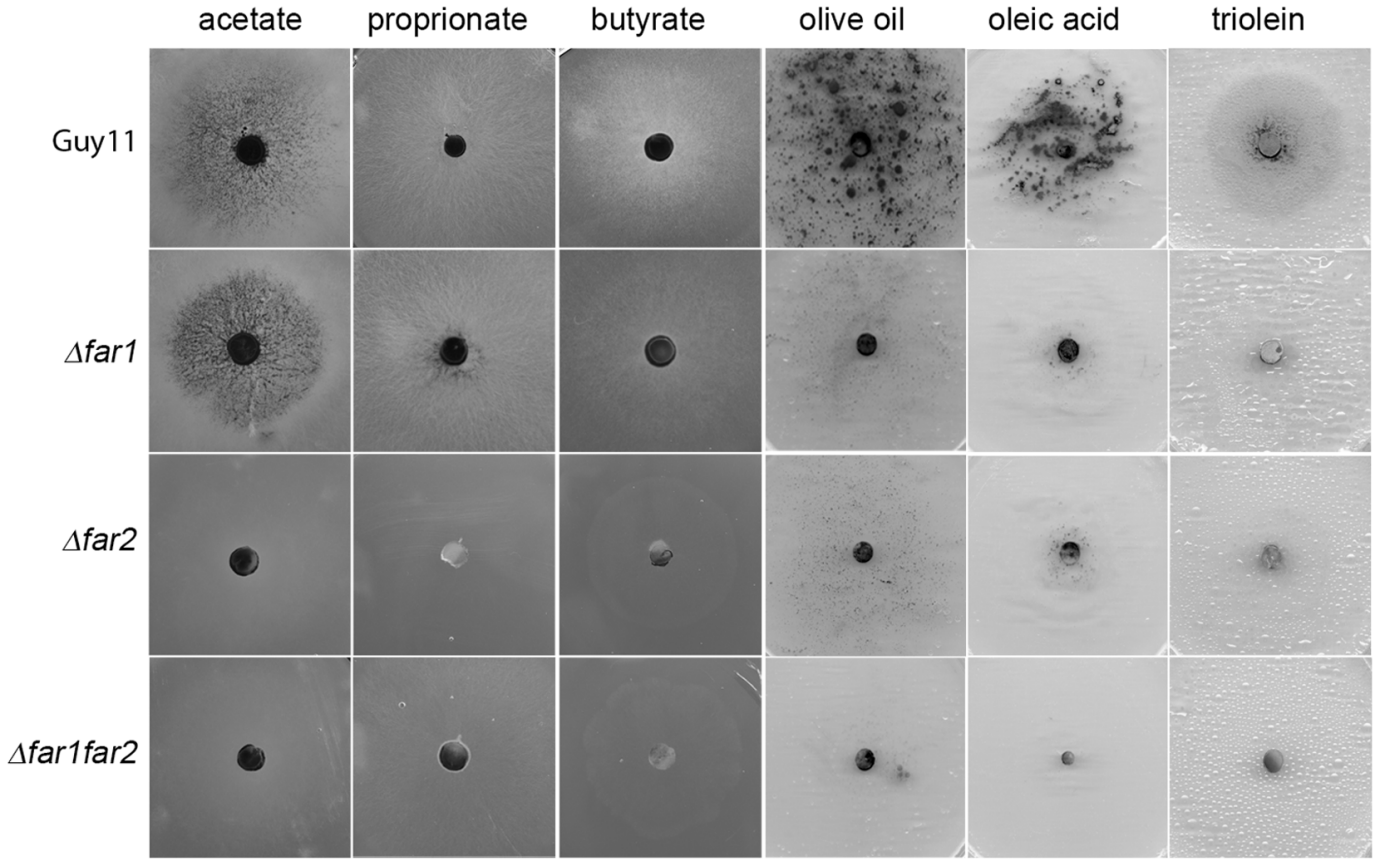
Photographs to show growth test of M. oryzae on differnet lipid sources. The wild type strain Guy11 and isogenic *Δfar1*, *Δfar2*, and *Δfar1Δfar2* double mutants were grown on minimal medium with fatty acids as the sole carbon sources. The following carbon sources were added to minimal medium; acetate (50 mM) propionate (0.1%), butyrate(10mM), olive oil (1%w/v), oleic acid (50mM) and triolein (50mM) in agar plates, inoculated with a *M.oryzae* mycelial plug and incubated at 24°C for 12 days.

To test the role of *FAR1* and *FAR2* in the regulation of lipid metabolism, *M.oryzae Δfar1*, Δ*far2* and Δ*far1*Δ*far2* mutants were grown on minimal medium supplemented with different lipids as sole carbon source, including both short and long-chain fatty acids, and also olive oil and the triglyceride, triolein. On short chain fatty acids, including butyrate (C3) and propionate (C4), the growth of the Δ*far1* mutant was similar to that of the wild type, while Δ*far2* mutants and the Δ*far1*Δ*far2* double mutant were unable to grow. By contrast, when grown on long chain fatty acids including decanoic (C10), dodecanoic (C12) and oleic (C18) acid, or on olive oil or triolein, the growth of both Δ*far1* and Δ*far2* mutants was severely reduced compared to Guy11 (Figure 4). The Δ*far1*Δ*far2* double mutant demonstrated growth defects similar to either of the single deletion mutants on these lipids.. Growth on other fatty acids was also tested including valeric (C5), myristic (C14) and palmitic (C16) acids, but neither Guy11 or any of the mutants was able to grow when these fatty acids were the sole carbon source (Table S2). We did not test C7 to C10 chain-length fatty acids which have been previously been shown to be toxic to fungi [18], [24]. We conclude that *FAR1* and *FAR2* therefore have overlapping but independent functions, with both regulators being necessary for growth on long chain fatty acids and triglycerides, but *FAR2* alone regulating growth of *M. oryzae* on short chain fatty acids.

### *FAR1* and *FAR2* Regulate Expression of Lipid Metabolic Genes in *M. oryzae*

**Figure 5:**
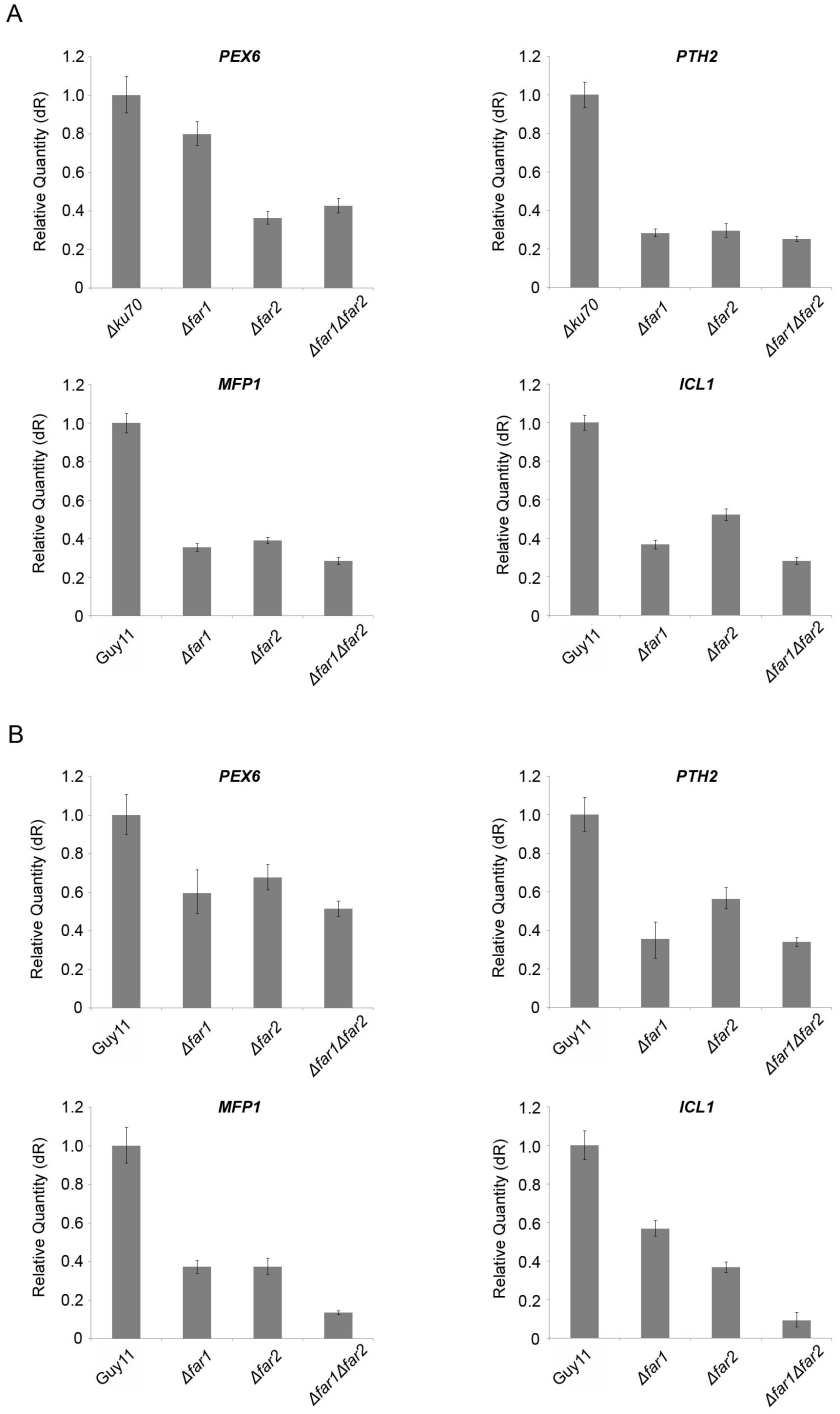
Bar charts to show gene expression profiles of *PEX6*, *PTH2*, *MFP1* and *ICL1* genes in *M. oryzae* mutants incubated in A. oleic acid and B. triolein as sole carbon source. Total RNA was extracted from fungal mycelium that was grown in complete medium for 48h and then transferred to minimal medium containing oleic acid or triolein for 24; *PEX6* (peroxisomal biogenesis), *MFP1* (fatty acid β-oxidation), *PTH2* (acetyl-CoA translocation), *ICL1* (glyoxylate cycle), were determined using quantitative real-time RT-PCR.

We next investigated whether *FAR1* and *FAR2* act as potential regulators of genes associated with lipid metabolism. To do this, we tested the expression of a range of genes associated with peroxisomal fatty acid β-oxidation and the glyoxylate cycle in *Δfar1* and *Δfar2* mutants. *MFP1* is a multi-functional protein required for fatty acid β-oxidation, *PEX6* is required for peroxisomal biogenesis, *PTH2* encodes a carnitine acetyl transferase involved in translocation of acetyl-CoA across the peroxisomal membrane, while *ICL1* encodes isocitrate lyase which catalyses the conversion of isocitrate to malate, the first step of the glyoxylate cycle. Total RNA was extracted from *Δfar1*, *Δfar2*, *Δfar1Δfar2* mutants and the Guy11 wild type strain of *M. oryzae*, that had been grown for 24 hours in either oleic acid or triolein as sole carbon source. The expression profiles of *MFP1*, *PEX6*, *PTH2* and *ICL1* genes were then determined, as shown in Figure 5. In the presence of oleic acid expression was significantly reduced for *PTH2* (70%) and *MFP1* (60–70 %) in *Δfar1*, *Δfar2*, and *Δfar1Δfar2* mutants. The expression of *ICL1* was also lower on oleic acid, with a 70% reduction in the Δ*far1*Δ*far2* mutant and a 60 % and 45 % reduction in Δ*far1* and Δ*far2* mutants, respectively. *PEX6* expression was also reduced, but much less so, particularly in the *Δfar1* mutant. when considered together, these results indicate that *FAR1* and *FAR2* are necessary for induction of genes associated with lipid substrate utilization.

### *FAR2* is Involved in Acetate Utilisation in *M. oryzae*

**Figure 6:**
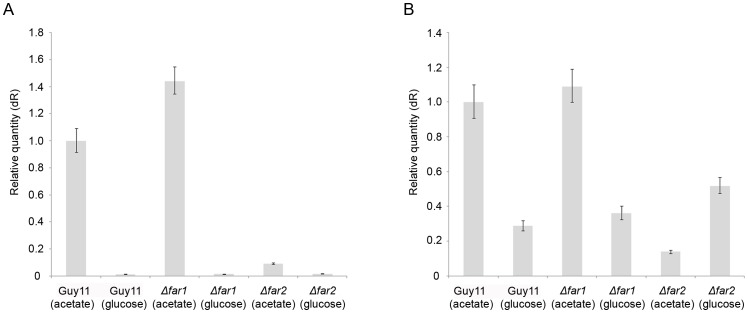
Bar charts to show gene expression profile of A. *ACS2* and B. *ACS3* acetyl CoA synthetase gene in Guy11 and the isogenic, *Δfar1* and *Δfar2* mutants. Total RNA was extracted from fungal mycelium that was grown in complete medium for 48h and then transferred to minimal medium containing oleic acid or triolein for 24*ACS2* and *ACS3* was determined using quantitative real-time RT-PCR.

We next tested whether Δ*far1*, Δ*far2* and Δ*far1*Δ*far2* mutants were able to utilise a range of carbon sources, including acetate as sole carbon source. We observed that while a Δ*far1* mutant is capable of growth on acetate, *Δfar2* mutants and the Δ*far1*Δ*far2* double mutant were unable to utilise acetate as a carbon source (Figure 4). This distinguishes *M. oryzae FAR2* from its *A. nidulans* homologue *farB,* which was able to grow normally on acetate [18]. To test how this regulation potentially operates we tested expression of acetyl-CoA synthetase genes in *M. oryzae*. Acetate is converted into acetyl-CoA through a reaction catalyzed by acetyl-CoA synthetase in the cytoplasm. Under conditions where either ethanol or acetate are the only available carbon source, expression levels of genes required for their utilisation are up-regulated [25], [26]. We therefore decided to determine whether expression acetyl-CoA synthetase genes was affected in *Δfar1* and *Δfar2* mutants. We identified *ACS2* and *ACS3*, which putatively encode acetyl-CoA synthetase and show significant similarity to *A. nidulans facA* [28]. Total RNA was obtained from *Δfar1* and *Δfar2* mutants and Guy11 grown in minimal medium with acetate or glucose as sole carbon source for 24 hours, and the expression profiles of *ACS2* and *ACS3* were determined. In the wild type strain, both *ACS2* and *ACS3* were highly up-regulated in the presence of acetate. *ACS2* expression was completely repressed on glucose, with *ACS3* showing only low-level expression. We observed that expression levels of *ACS2* and *ACS3* were similar in the acetate-utilizing *Δfar1* mutant with normal glucose repression. By contrast, in the *Δfar2* mutant there was no significant induction of *ACS2* or *ACS3* in the presence of acetate (Figure 6). We conclude that *FAR2* plays a wider role in both lipid and acetate metabolism in *M. oryzae*.

## Discussion

The mobilisation of lipid reserves is of fundamental importance to the pathogenic lifestyle of the rice blast fungus *M. oryzae* [1]–[4]. Lipid bodies, which are abundant in spores of the fungus, are transported to the incipient appressorium where they are rapidly degraded at the onset of turgor generation and plant infection [4]. Lipolysis in the appressorium leads to β-oxidation of fatty acids, generating acetyl CoA, which is metabolized via the glyoxylate shunt bypass enabling gluconeogenesis [11]–[14], but is also a metabolic intermediate necessary for synthesis of melanin, isoprenoids, amino acids, and a range of secondary metabolites. The pool of acetyl CoA in the appressorium of *M. oryzae* is therefore fundamental to the generation of structural components required for cellular remodelling as the infection cell prepares to apply the tremendous mechanical force required to breach the rice cuticle and undergo rapid polarised growth into underlying epidermal cells [1]–[4].

The primary objective of this study was to identify and characterise factors responsible for regulation of genes involved in lipid metabolism in *M. oryzae*. The FarA and FarB proteins, previously identified in *A. nidulans,* regulate fatty acid catabolism and peroxisome function [18]. We identified closely-related genes, *FAR1* and *FAR2*, in the *M. oryzae* genome, which are highly conserved among filamentous ascomycetes and which encode proteins containing Zn_2_-Cys_6_ binuclear DNA binding domains. Zn_2_-Cys_6_ binuclear cluster DNA binding proteins are exclusive to fungi [27] and, with few exceptions, are constitutively expressed in the nucleus with transcriptional activation regulated at the level of DNA binding [28]. We found that *FAR1* and *FAR2* are also constitutively expressed and localised to the nucleus in *M. oryzae*, consistent with characteristics of this class of transcriptional regulator.

Targeted deletion of *FAR1* and *FAR2* resulted in phenotypes consistent with their involvement in regulation of fatty acid utilisation, but also suggested some functional differences to the previously characterised *A. nidulans farA* and *farB*. We found, for instance, that *Δfar1, Δfar2* and Δ*far1*Δ*far2* mutants are unable to grow on long chain fatty acids and olive oil (C12 and above), as well as triglycerides such as triolein. In *M. oryzae* both *FAR1* and *FAR2* therefore appear to be independently required for utilisation of long chain fatty acids. By contrast, in *A. nidulans* only *farA* is necessary to utilise long chain fatty acids whilst *farB* mutants grow normally, but are unable to utilise short chain fatty acids such as butyrate [18]. In *M. oryzae*, *Δfar1* mutants can utilise short chain fatty acids such as propionate (C3) and butyrate (C4), but *Δfar2* mutants are unable to grow on any short chain fatty acids. In *M. oryzae, FAR2* is therefore essential for growth on both long chain and short chain fatty acids, rather than having a more restricted regulatory function like FarB in *A. nidulans* [19]. Consistent with their role in lipid metabolism, deletion of *FAR1* and *FAR2* significantly affected expression of genes involved in fatty acid β-oxidation (*MFP1*), acetyl-CoA translocation (*PTH2*), peroxisomal biogenesis (*PEX6*), and the glyoxylate cycle (*ICL1*). When compared to the wild type strain, expression of *MFP1*, *PTH2*, *PEX6*, and *ICL1* were considerably lower under conditions that normally induce gene expression. Far1 and Far2 are therefore likely to be wide domain regulators of a range of gene functions associated with lipolysis, fatty acid β-oxidation, peroxisome function and gluconeogenesis.

The identification of *farA* and *farB* in *A. nidulans* was achieved by screening for mutants specifically affected in butyrate utilization but capable of growth on glutamate and acetate [19]. However, in *M. oryzae Δfar2* mutants are unable to grow on acetate, suggesting an additional role for *FAR2* in regulation of acetate utilisation in *M. oryzae*, again in contrast to *farB*. In *A. nidulans* a second transcriptional activator, encoded by the *facB* gene, has been shown to be necessary for regulation of acetate metabolism [29]. Interestingly, *M. oryzae* also has an orthologue of facB, but its role has yet to be determined. Growth on acetate depends on acetyl-CoA synthetase-dependent formation of acetyl-CoA in the cytosol. The conversion of acetyl-CoA to acetyl-carnitine by a cytosolic acetyl-carnitine transferase and transported into the mitochondrion, where it is converted back to acetyl-CoA by a mitochondrial acetyl-carnitine transferase for metabolism by the tricarboxylic acid cycle. The expression of *ACS2* and *ACS3* encoding acetyl-CoA synthetases in *M. oryzae* were, as expected, acetate-inducible and glucose-repressible in the wild type strain, and also in the *Δfar1* mutant. However in a *Δfar2* mutant, no significant induction of *ACS2* or *ACS3* was seen on acetate, consistent with its failure to grow on acetate as sole carbon source. In *A. nidulans*, addition genes encoding acetyl-CoA synthetase (*facA)*, and cytosolic acetyl-carnitine transferase (*facC*), are induced by acetate via the FacB activator [30], [31]. In *S. cerevisae* growth on ethanol or acetate as sole carbon sources is dependent on the Cat8, Sip4, and Adr1 activators as well as the Snf1 kinase [32], while in the presence of glucose the Mig1 repressor represses the expression of these genes [33].

Previous studies have shown that fatty acid β-oxidation, peroxisomal biogenesis, acetyl-CoA translocation and the glyoxylate cycle are all involved in the ability of *M. oryzae* to cause rice blast disease [11], [12], [14]. *M. oryzae Δpex6* and *Δpth2* mutants, for example, have been shown to be non-pathogenic, while *Δmfp1* and *Δicl1* mutants display reduced and delayed infection, respectively [11]–[14]. Furthermore, the Ech1 enoyl-CoA hydratase, a mitochondrial β-oxidation enzyme essential for growth on C16 and C18 fatty acids, is involved in appressorium function and virulence [34]. Interestingly, we found that Δ*far1*, Δ*far2* and Δ*far1*Δ*far2* double mutants were not impaired in lipid body mobilisation to the appressorium, or subsequent lipolysis during turgor generation in the appressorium. This study therefore provides evidence that lipid body mobilisation during appressorium development by *M. oryzae*, which occurs on the rice leaf surface in the absence of exogenous nutrients and is dependent on autophagy [4], [7], [9], [10], is regulated separately from lipid utilization and independently of Far1 and Far2.

## Materials and Methods

### Strains, Culture Conditions and DNA Analysis

All strains of *M. oryzae* are stored in the laboratory of NJT, University of Exeter. Standard procedures for fungal growth, maintenance, nucleic acid extraction and transformation were performed as described previously [35]. Minimal medium was supplemented with 1% (w/v) glucose or 1% (w/v) olive oil as a sole carbon source. Other alternative carbon sources used were acetate, oleic acid and triolein (50 mM), butyrate and valeric (10mM), propionate (0.1%), decanoic, myristic, palmitic and dodecanoic acid (2.5 mM). The pH was adjusted to 6.5 and 0.5% tergitol was used to help disperse long chain fatty acids. Gel electrophoresis, restriction enzyme digestion, DNA gel blot hybridization and sequencing were performed using standard procedures [36].

### Identification and Targeted Gene Disruption of *FAR1* and *FAR2*

*FAR1* and *FAR2* genes were identified by homology to *A. nidulans FarA* and *FarB* genes obtained from the *A. nidulans* genome database. Both genome databases were retrieved from the Broad Institute (Massachusetts Institute of Technology, Cambridge, MA). (www.broad.mit.edu/annotation/fungi/) and used to design gene specific primers (Table S1). Targeted gene deletion of the *M. oryzae FAR1* and *FAR2* was performed by the split marker strategy [37] Vectors were constructed using a hygromycin B selectable marker *hph* or the Basta resistant *BAR* gene [38] and transformed into the wild type Guy11. Transformants were selected in the presence of hygromycin (200 µg ml^-1^) and for the double gene deletions, glufosinate ammonium (50 µg ml^-1)^ (Sigma) was used. Mutants were confirmed by DNA gel blot analysis [36].

### Assays for Infection-related Morphogenesis

Rice infection assays were performed as described previously [35]. Conidial suspensions were diluted in 0.2% gelatine to 1×10^4^ conidia ml^-1^ for rice infections using the dwarf Indica rice cv. CO-39. Conidia were sprayed onto 14-day-old seedlings using an artist’s airbrush. Following spray-inoculation, plants were placed in polythene bags and incubated in a controlled environment chamber at 24 °C with a 12 hour light-12 hour dark period and 90% relative humidity. After 48 hours the polythene bags were removed and plants incubated for 2–3 days for disease symptoms to develop.

### Cytological Analysis

Lipid droplets in germinating conidia and appressoria were visualized by staining with Bodipy 493/503 (Invitrogen). Conidia were harvested, washed and re-suspended in sterile distilled water to a concentration of 2×10^5^ spores mL^-1^. Conidia were inoculated onto plastic cover slips in a moist chamber at 24 °C and observed for appressorium formation and lipid mobilization at intervals, by mounting directly in fresh Bodipy 493/503 solution for 15 min.

### Generation of *FAR1-GFP* and *FAR2-GFP* Gene Fusions

The *FAR1-GFP* gene fusion construct was made by amplifying a 5.0 kb fragment of *FAR1* containing 1.4 kb of upstream promoter sequence using the primers FarA.SpeI.F3 and FarA.EcoRI.R2 (Table S1) creating *Spe*I and *Eco*RI sites at the fragment. The amplicon was digested and cloned into pCB1532 [38], which carries a selectable marker bestowing resistance to sulfonylurea. s*GFP*-*trpC* was amplified using the primers GFP.TrpC.EcoRI.F1 and TrpC.XhoI.R creating *Eco*RI and *Xho*I restriction sites at the end of the fragment. The amplicon was digested and sub-cloned in-frame with *FAR1* to create *FAR1-GFP*. The *FAR2-GFP* gene fusion construct was made using recombination mediated PCR directed plasmid construction *in vivo* in yeast [39]. In this technique, 1284 pNEB-Nat-Yeast cloning vector was used which contains the *URA3* gene, allowing uracil synthesis and therefore complementation of uracil (-) auxotrophy. *Sac*I and *Hin*dIII were used to linearize the yeast cloning vector. The sulfonylurea resistance gene, *FAR2* open reading frame, including promoter, and *GFP*-*trpC* were amplified separately using primers listed in Table S1. Primers used to amplify the PCR fragments were designed to incorporate overhangs corresponding to the adjacent PCR fragments or to the yeast plasmid. Homologous recombination resulted in assembly of the fragments in the correct orientation to generate the gene fusion construct. To screen for correct clones, yeast was grown on minimal medium. For large-scale DNA preparation, the plasmid was transformed into *E. coli* before plasmid DNA extraction for fungal transformation. Both *FAR1-GFP* and *FAR2-GFP* were transformed into the wild type strain Guy11.

### Light and Epifluorescence Microscopy

Epifluorescence microscopy to visualize GFP and stained samples was routinely carried out using an IX81 motorized inverted microscope (Olympus) equipped with a UPlanSApo 100X/1.40 Oil objective (Olympus). Excitation of fluorescently-labelled proteins and lipid droplets was carried out using a VS-LMS4 Laser-Merge-System with solid-state lasers (488 nm/50 mW). The laser intensity was controlled by a VS-AOTF100 System and coupled into the light path using a VS-20 Laser-Lens-System (Visitron System). Images were captured using a Charged-Coupled Device camera (Photometric CoolSNAP HQ2, Roper Scientific). All parts of the system were under the control of the software package MetaMorph (Molecular Devices).

### Gene Expression Analysis

Total RNA was extracted from *M. oryzae* strains grown on minimal medium supplemented with either oleic acid, triolein, glucose or acetate using lithium chloride as described previously [40] and was treated with DNaseI (Invitrogen). Single stranded cDNA was synthesized using AffinityScript cDNA master mix (Agilent Technologies), and qPCR reactions were set up using the Brilliant II SYBR Green Q-PCR master mix (Agilent Technologies), according to the manufacturer’s instructions. The primers pairs used to amplify probes for the relevant genes to determine the relative expression of are listed in Table S1. All reactions were carried out in triplicate for each cDNA sample. The *M. oryzae* β-tubulin gene (MGG_00604.6) was used as an endogenous control and Guy11 grown in oleic acid or triolein cDNA was used as the calibrator. Reactions (25 µL) were analysed on an Mx3005P QPCR System, running MxPro QPCR Software (Agilent Technologies), for 40 cycles following standard protocols. Expression analysis was performed by determining the Ct value of each gene and normalised by the Ct value of β-tubulin.

### Multiple Sequence Alignment

Multiple sequence alignment was created using ClustalW ([41] ) and conserved residues highlighted using BoxShade (http://www.ch.embnet.org/software/BOX_form.html).

### Phylogenetic Tree

Protein sequences obtained from Genbank (http://www.ncbi.nlm.nih.gov/) and Broad Institute (http://www.broadinstitute.org/scientific-community/science/projects/fungal-genome-initiative/fungal-genome-initiative) were aligned using MUSCLE ([42]). Conserved regions of the alignment were sampled using GBlocks ([43]) and a maximium likelihood phylogenetic tree created using PhyML with 100 bootstraps ([44]).

### Supersage Gene Expression Data

Gene expression data, generated using Supersage, from the appressorium development of *M. oryzae* (relative to mycelium grown in rich medium) was obtained from a previously published study ([45]) and used to make a heatmap with the statistical package R (http://www.r-project.org/).

## Supporting Information

Figure S1 Predicted amino acid sequence of the *FAR1* gene product. Sequences were aligned using the program CLUSTALW. Identical amino acids are highlighted on a black background and similar amino acids on a light grey background. Gaps in the alignment are indicated by dashes. Sequences aligned were the predicted products of *FAR1* (MGG_01836.6), *A. nidulans* FarA (AN7050.2), *N. crassa* cutinase transcription factor alpha (NCU08000.5) and *F.oxysporum* Ctf1 (FOXG_04196.2). Sequences in red show the Zn_2_Cys_6_ binuclear cluster domain while sequences in blue show the fungal specific transcription factor domain.
(DOCX)

Figure S2 Predicted amino acid sequence of the *FAR2* gene product. Sequences were aligned using the program CLUSTALW. Identical amino acids are highlighted on a black background and similar amino acids on a light grey background. Gaps in the alignment are indicated by dashes. Sequences aligned were the predicted products of *FAR2* (MGG_08199.6), *A. nidulans FarB* (AN1425.2), *N. crassa* cutinase transcription factor beta (NCU03643.5) and *F.oxysporum* cutinase transcription factor 1 beta (FOXG_01610.2). Sequences in red show the Zn_2_Cys_6_ binuclear cluster domain while sequences in blue show the fungal specific transcription factor domain.
(DOCX)

Figure S3 Targeted gene deletion of **A**. *FAR1* and **B**. *FAR2* using the split marker technique. **C.** DNA gel blot analysis of putative Δ*far1* mutants digested with *Mfe* I and probed with 1.4 kb of the *FAR1* for presence and absence of the coding region of the gene. **D.** DNA gel blot analysis of putative Δ*far1* mutants digested with *Mfe* I, probed with a 1 kb fragment of 5’-UTR to identify Δ*far1* mutants based on a size difference caused by the insertion of the selectable marker at the *FAR1* locus. Transformants A1 and A2 were chosen as putative Δ*far1* mutants **E.** DNA gel blot analysis of putative Δ*far2* and Δ*far1*Δ*far2* mutants probed with 1.4 kb of the coding sequence of *FAR2* for presence and absence of the coding region. **F.** DNA gel blot analysis of putative Δ*far2* mutants probed with 1.2 kb of hygromycin cassette for the presence of the hygromycin resistant fragment. Transformants B1, B2 and B3 were chosen as putative Δ*far2* mutants. **G.** DNA gel blot analysis of putative Δ*far1*Δ*far2* double mutants probed with 1.2 kb of BASTA cassette for the presence of BAR resistant fragment. Transformants D1, D2 and D4 were chosen as putative Δ*far1*Δ*far2* double mutants.
(TIF)

Figure S4 Epifluorescence micrographs to show distribution of lipid droplets during appressorium morphogenesis in *Δfar1* and *Δfar2* mutants of *M. oryzae* There were no apparent differences shown by the mutants compared to the isogenic wild type Guy11. Scale bar = 10 µm.
(TIFF)

Figure S5 Bar charts to show quantitative analysis of lipid body distribution during infection related development by *M. oryzae*. Conidia were allowed to germinate in water drops on the surface of cover slips and to undergo infection related development. Samples were removed at intervals over an 8 hour period and stained for the presence of triacylglycerol by using Bodipy stain. The percentage of fungal structures that contained lipid bodies at a given time was recorded from a sample of 100 germinated conidia. The bar charts show the mean and standard deviation from 2 independent replications of the experiment. **A.** Wild type strain, Guy11. **B.** *Δfar1* mutant; **C.** *Δfar2* mutant; **D.** *Δfar1Δfar2* mutant.
(TIFF)

Figure S6 Bar charts to show quantitative analysis of lipid distribution during infection related development by *M. oryzae*. Conidia were allowed to germinate in water drops on the surface of cover slips and to undergo infection related development. Samples were removed at intervals over an 8 hour period and stained for the presence of triacylglycerol by using Bodipy stain. The percentage of fungal structures that contained lipid bodies at a given time was recorded from a sample of 100 germinated conidia. The bar charts show the mean and standard deviation from 2 independent replications of the experiment. **A.** Wild type strain, Guy11; **B.** *Δatg8* autophagy mutant.
(TIFF)

Table S1 Sequences of oligonucleotide primers used in this study.
(DOCX)

Table S2 Growth of *M. oryzae* Δ*far1*, Δ*far2* and Δ*far1*Δ*far2* mutants on fatty acids as sole carbon sources.
(DOCX)
