# Supplementary figures and images for: FAR1 and FAR2 Regulate the Expression of Genes Associated with Lipid Metabolism in the Rice Blast Fungus Magnaporthe oryzae
Source: PLoS One. 2014 Jun 20;9(6):e99760. doi: 10.1371/journal.pone.0099760 (PMC4064970; doi:10.1371/journal.pone.0099760)

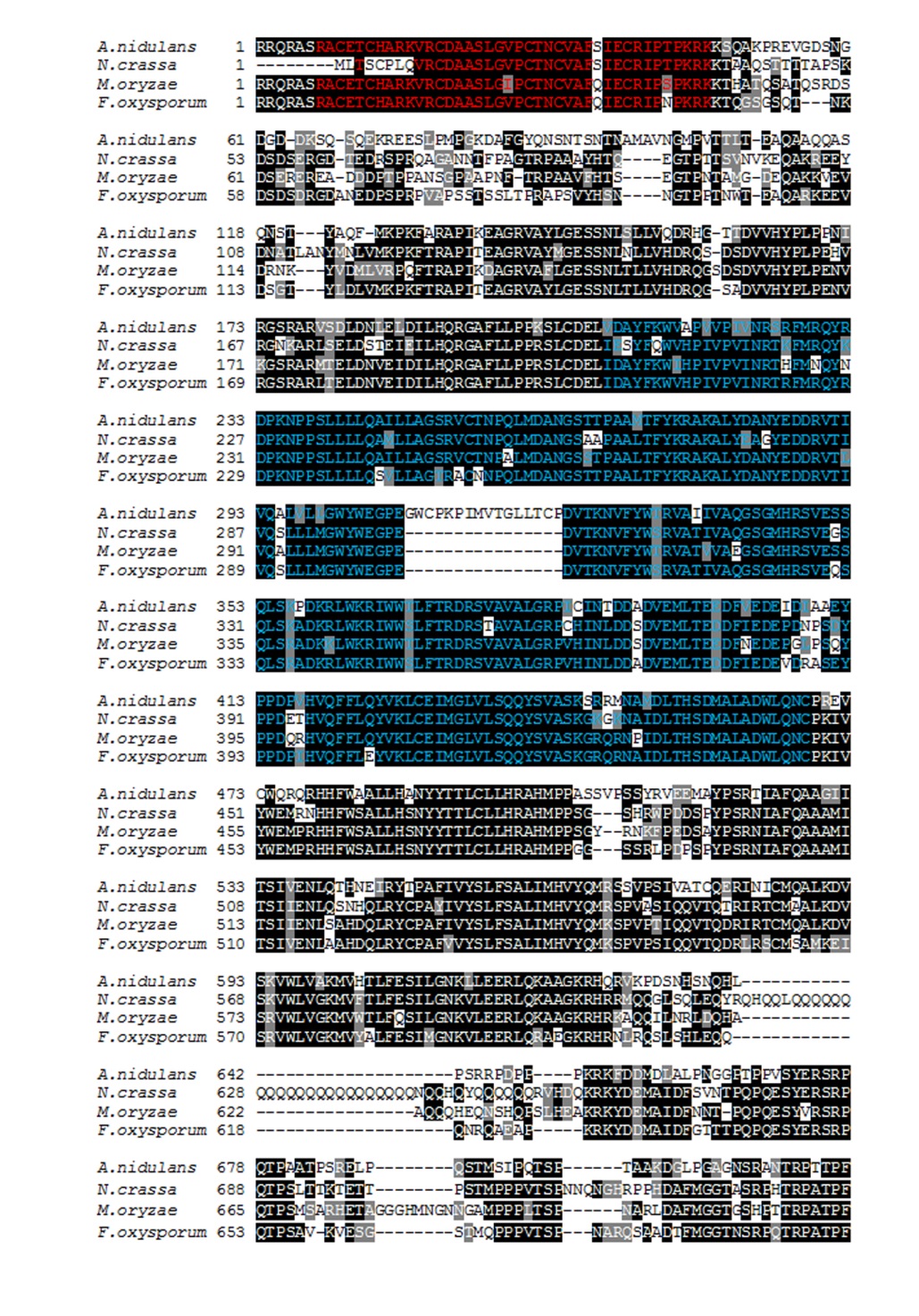


**Supplementary Figure 1.**

Supplement: Figure S1 — Predicted amino acid sequence of the FAR1 gene product. Sequences were aligned using the program CLUSTALW. Identical amino acids are highlighted on a black background and similar amino acids on a light grey background. Gaps in the alignment are indicated by dashes. Sequences aligned were the predicted products of FAR1 (MGG_01836.6), A. nidulans FarA (AN7050.2), N. crassa cutinase transcription factor alpha (NCU08000.5) and F.oxysporum Ctf1 (FOXG_04196.2). Sequences in red show the Zn2Cys6 binuclear cluster domain while sequences in blue show the fungal specific transcription factor domain. (DOCX) [file pone.0099760.s001.docx]

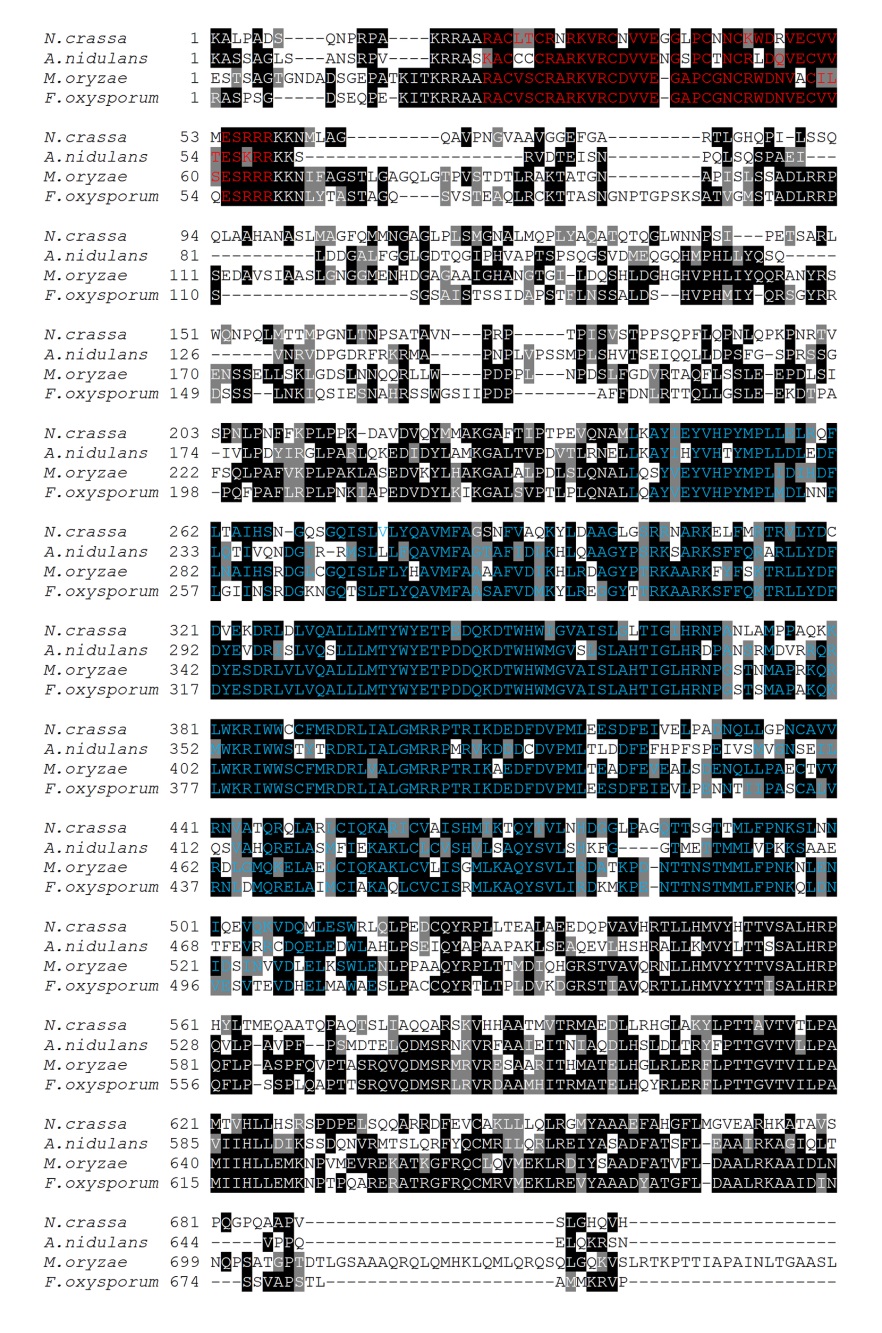


**Supplementary Figure 2.**

Supplement: Figure S2 — Predicted amino acid sequence of the FAR2 gene product. Sequences were aligned using the program CLUSTALW. Identical amino acids are highlighted on a black background and similar amino acids on a light grey background. Gaps in the alignment are indicated by dashes. Sequences aligned were the predicted products of FAR2 (MGG_08199.6), A. nidulans FarB (AN1425.2), N. crassa cutinase transcription factor beta (NCU03643.5) and F.oxysporum cutinase transcription factor 1 beta (FOXG_01610.2). Sequences in red show the Zn2Cys6 binuclear cluster domain while sequences in blue show the fungal specific transcription factor domain. (DOCX) [file pone.0099760.s002.docx]

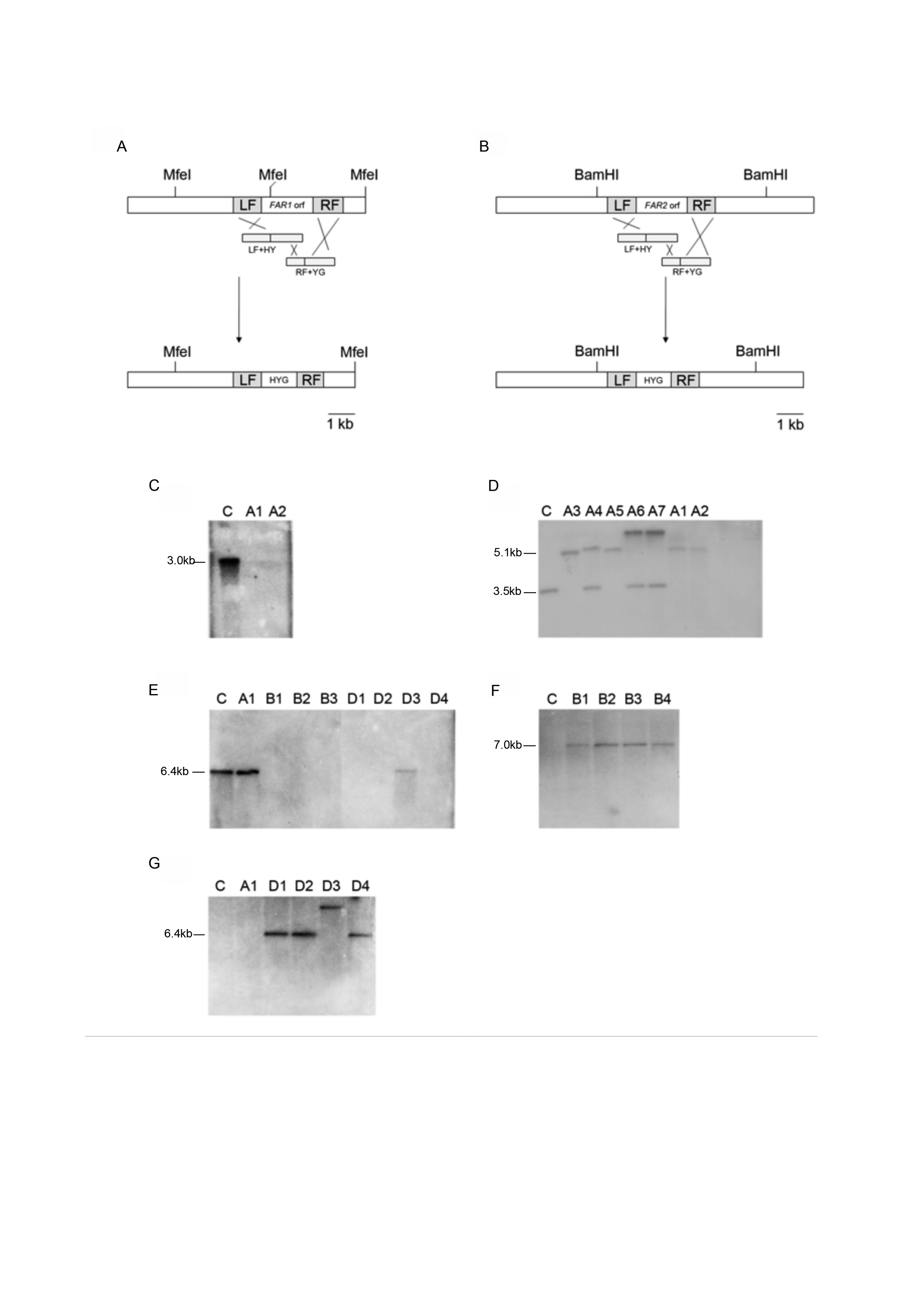

Supplement: Figure S3 — Targeted gene deletion of A. FAR1 and B. FAR2 using the split marker technique. C. DNA gel blot analysis of putative Δfar1 mutants digested with Mfe I and probed with 1.4 kb of the FAR1 for presence and absence of the coding region of the gene. D. DNA gel blot analysis of putative Δfar1 mutants digested with Mfe I, probed with a 1 kb fragment of 5’-UTR to identify Δfar1 mutants based on a size difference caused by the insertion of the selectable marker at the FAR1 locus. Transformants A1 and A2 were chosen as putative Δfar1 mutants E. DNA gel blot analysis of putative Δfar2 and Δfar1Δfar2 mutants probed with 1.4 kb of the coding sequence of FAR2 for presence and absence of the coding region. F. DNA gel blot analysis of putative Δfar2 mutants probed with 1.2 kb of hygromycin cassette for the presence of the hygromycin resistant fragment. Transformants B1, B2 and B3 were chosen as putative Δfar2 mutants. G. DNA gel blot analysis of putative Δfar1Δfar2 double mutants probed with 1.2 kb of BASTA cassette for the presence of BAR resistant fragment. Transformants D1, D2 and D4 were chosen as putative Δfar1Δfar2 double mutants. (TIF) [file pone.0099760.s003.tif]

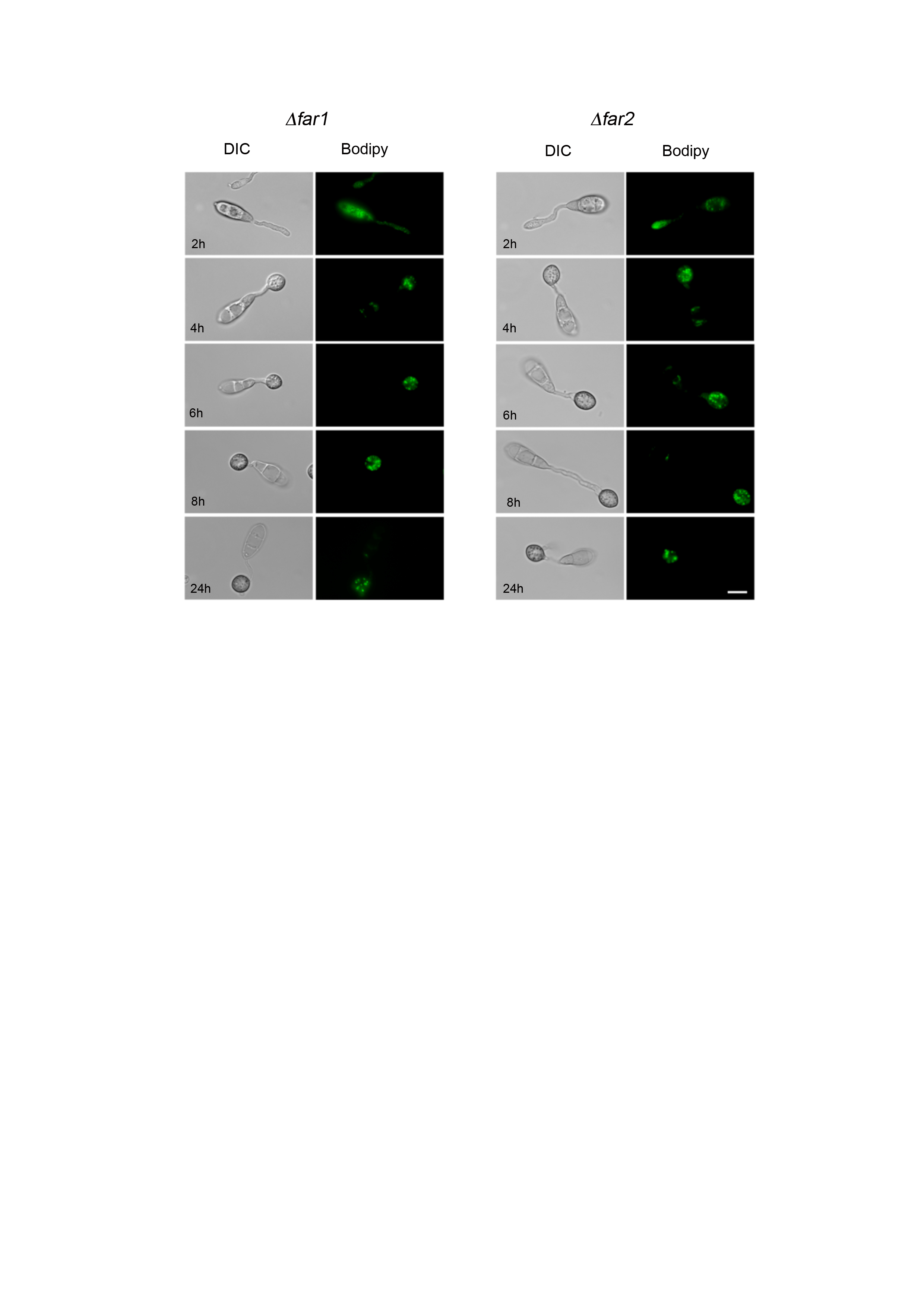

Supplement: Figure S4 — Epifluorescence micrographs to show distribution of lipid droplets during appressorium morphogenesis in Δfar1 and Δfar2 mutants of M. oryzae There were no apparent differences shown by the mutants compared to the isogenic wild type Guy11. Scale bar = 10 µm. (TIFF) [file pone.0099760.s004.tiff]

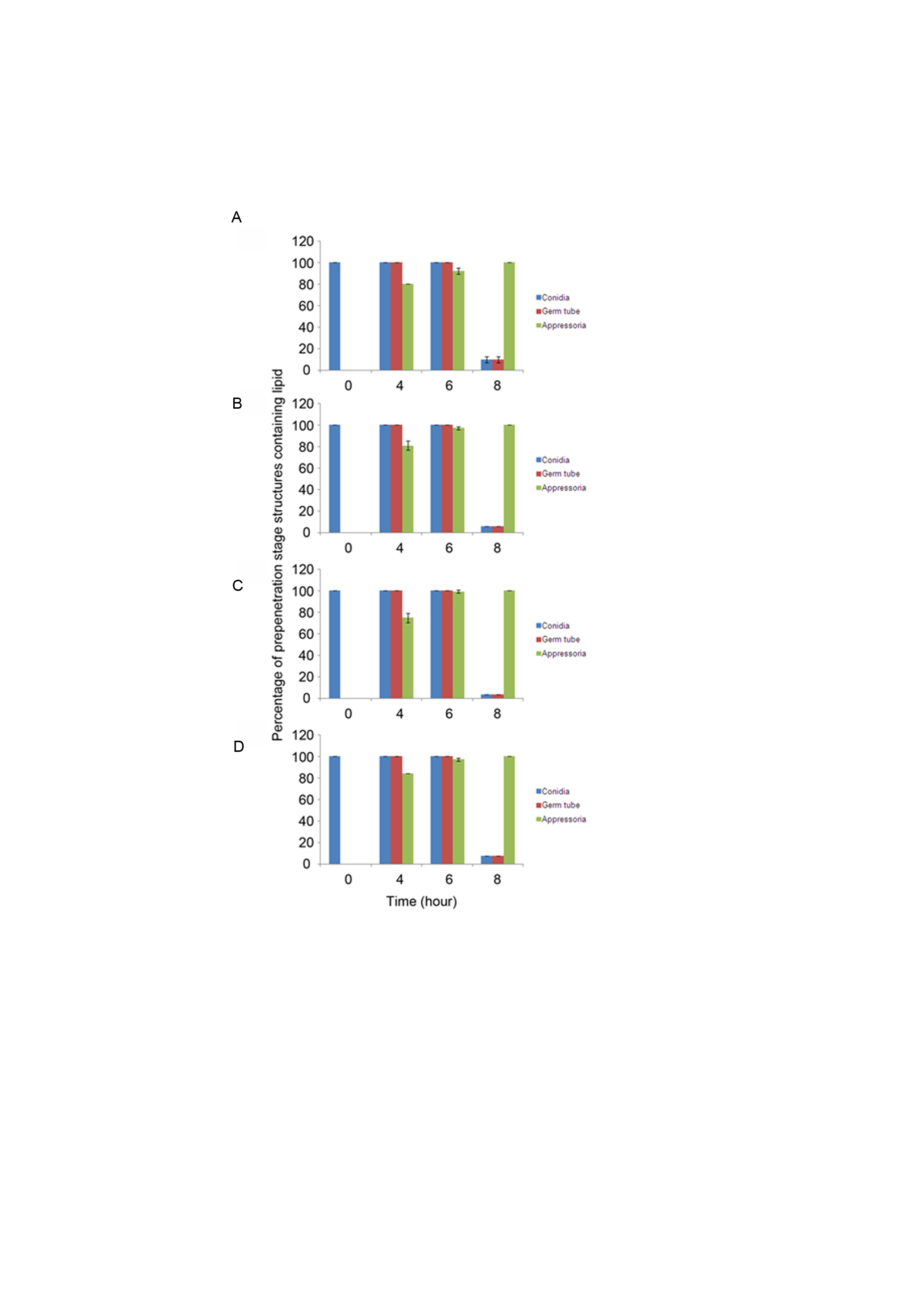

Supplement: Figure S5 — Bar charts to show quantitative analysis of lipid body distribution during infection related development by M. oryzae. Conidia were allowed to germinate in water drops on the surface of cover slips and to undergo infection related development. Samples were removed at intervals over an 8 hour period and stained for the presence of triacylglycerol by using Bodipy stain. The percentage of fungal structures that contained lipid bodies at a given time was recorded from a sample of 100 germinated conidia. The bar charts show the mean and standard deviation from 2 independent replications of the experiment. A. Wild type strain, Guy11. B. Δfar1 mutant; C. Δfar2 mutant; D. Δfar1Δfar2 mutant. (TIFF) [file pone.0099760.s005.tiff]

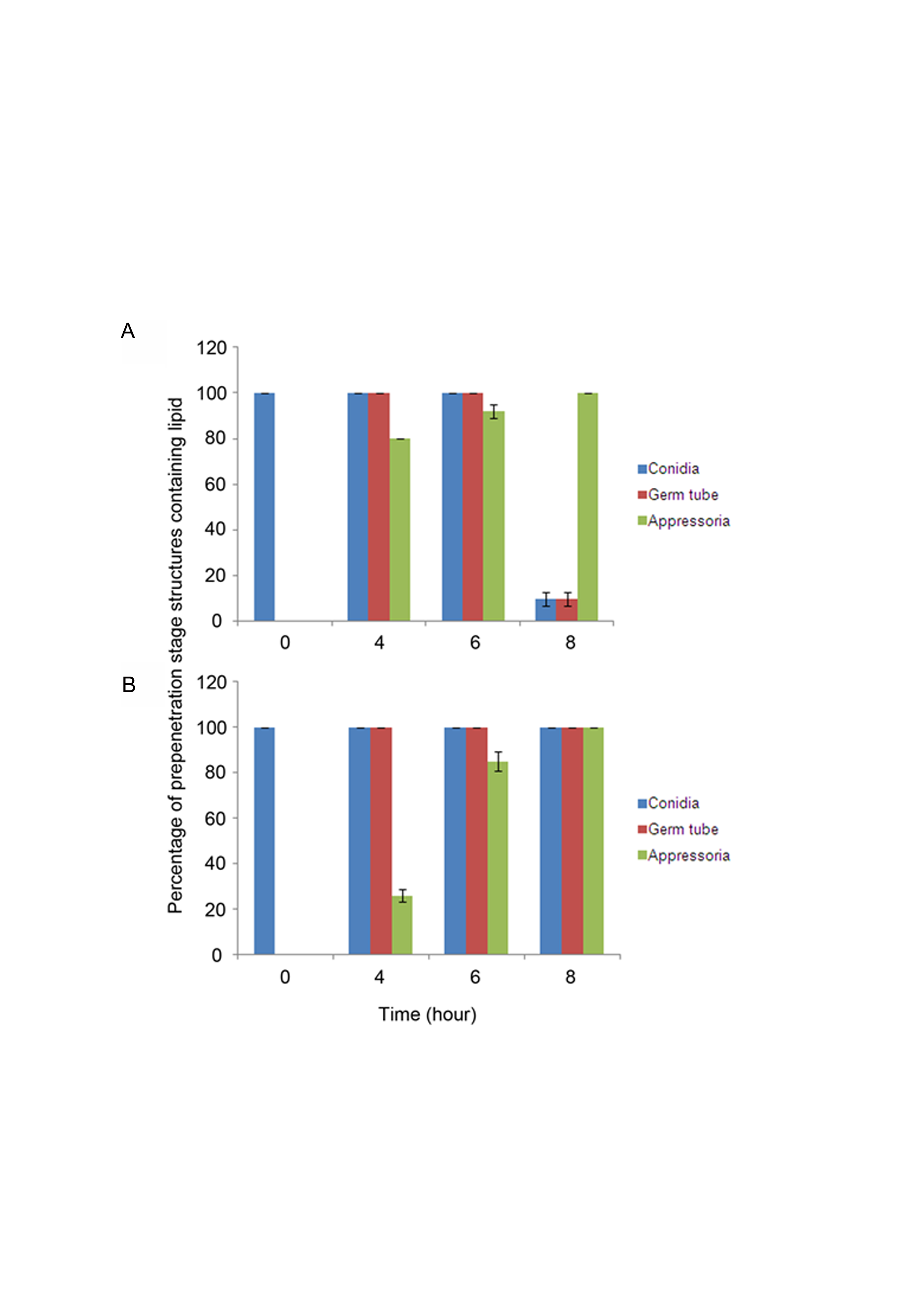

Supplement: Figure S6 — Bar charts to show quantitative analysis of lipid distribution during infection related development by M. oryzae. Conidia were allowed to germinate in water drops on the surface of cover slips and to undergo infection related development. Samples were removed at intervals over an 8 hour period and stained for the presence of triacylglycerol by using Bodipy stain. The percentage of fungal structures that contained lipid bodies at a given time was recorded from a sample of 100 germinated conidia. The bar charts show the mean and standard deviation from 2 independent replications of the experiment. A. Wild type strain, Guy11; B. Δatg8 autophagy mutant. (TIFF) [file pone.0099760.s006.tiff]
