# Supplementary material for: FAR1 and FAR2 Regulate the Expression of Genes Associated with Lipid Metabolism in the Rice Blast Fungus Magnaporthe oryzae
Source: PLoS One. 2014 Jun 20;9(6):e99760. doi: 10.1371/journal.pone.0099760 (PMC4064970; doi:10.1371/journal.pone.0099760)
Supplement: Table S1 — Sequences of oligonucleotide primers used in this study. (DOCX) [file pone.0099760.s007.docx]

**Supplementary Table 1.** Sequences and primers used in this study

| Primer name | Sequence 5’-3’ |
| --- | --- |
| Deletion of *FAR1* gene  FarA50.1  M13F  FarA30.1  M13R | ACATTCAGGTAGGGAGGACACAAA  GTCGTGACTGGGAAAACCCTGGCGCGTCTGCTCCTTCGCGCCATTGTT  TCCTGTGTGAAATTGTTATCCGCTACAGAGGAGGACTTCAACGAGGAC  CTGGGAATAGTTGATCGGGCTGAA |
| Deletion of *FAR2* gene  FarB50.1  M13F  FarB30.2  M13R | CATAACCTGTCTTTCTGCCTACCT  GTCGTGACTGGGAAAACCCTGGCGCCGCTCGTCGTTTTGTGATCTTGG  TCCTGTGTGAAATTGTTATCCGCTGTAGCACAAACATGGCTCCTCGTA  GGTTTGGTCCTCAGGCTCACTTTC |
| Amplification of *MoFAR1* fragment  FarA.SpeI.F3  FarA.EcoRI.R2 | CGACTAGTTGGACTGATACTTGGCGTGG  CGGAATTCAACAAGTGCAGAAAAGTCGATAT |
| Amplification of *MoFAR2* fragment  ProFarB.F1  FarB.R4 | GATTATTGCACGGGAATTGCATGCTCTCACTACCTACCTACCTAATACTCC  AGACGATACAGCCACCTGC |
| Amplification of GFP-*TrpC* fragment  GFP.TrpC.EcoRI.F1  TrpC.XhoI.R  FarBGFP.F1  FarBGFP.R1 | CGGAATTCATGGTGAGCAAGGGCGAGG  ATCTCGAGGTGGAGATGTGGAGTGGGCGC  AATAACGCACAGCAGGTGGCTGTATCGTCTATGGTGAGCAAGGGCGAGG  TTCACACAGGAAACAGCTATGACCATGATTAGTGGAGATGTGGAGTGGG |
| Amplification of SUR  Resistance gene  SUR.F  SUR.R | AACTGTTGGGAAGGGCGATCGGTGCGGGCCGTCGACGTGCCAACGCCA  GTCGACGTGAGAGCATGCA |
| Amplification *PEX6* probe  1Fq.Pex6  2Rq.PEX6 | AAATTCACACTCCACCCTTCA  GGCACTCACACTGGGAATC |
| Amplification of *PTH2* probe  1Fq.PTH  2Rq. PTH | CGAGTACATCACAGCCGCT  CGATGTTGAAGTTGATGCTGT |

**Supplementary Table 1 (contd)** Sequences and primers used in this study

| Primer name | Sequence 5’-3’ |
| --- | --- |
| Amplification of *MFP1* probe  Fq.MFP1  Rq.MFP1 | TGTCTATGAGGGAGCCGAG  ACCCCCTCAGGAAGATGCT |
| Amplification of *ICL1* probe  Fq.ICL1  Rq.ICL1 | GTGTACCCCGAGCAAAAACT  ACCCGTCACCATCTTCTGC |
| Amplification of *ACS2* probe  Fq.ACS2  Rq.ACS2 | TGACGACCACGCCGAACAA  CCGCATCCTCAACCGTAGA |
| Amplification of *ACS3* probe  Fq.ACS3  Rq.ACS3 | TCAGAGGCAGAGTGGACGA  CCAATGCTACGCCGAACCT |
