# Supplementary material for: FAR1 and FAR2 Regulate the Expression of Genes Associated with Lipid Metabolism in the Rice Blast Fungus Magnaporthe oryzae
Source: PLoS One. 2014 Jun 20;9(6):e99760. doi: 10.1371/journal.pone.0099760 (PMC4064970; doi:10.1371/journal.pone.0099760)
Supplement: Table S2 — Growth of M. oryzae Δfar1, Δfar2 and Δfar1Δfar2 mutants on fatty acids as sole carbon sources. (DOCX) [file pone.0099760.s008.docx]

**Supplementary Table 2**. Growth of *M. oryzae* *Δfar1, Δfar2,* and *Δfar1/Δfar2* mutants on fatty acids as the sole carbon sources.

| Carbon source | Guy11 | *Δfar1* | *Δfar2* | *Δfar1/far2* |
| --- | --- | --- | --- | --- |
| Acetate (C2) | +++++ | +++++ | + | + |
| Propionate (C3) | ++++ | ++++ | + | + |
| Butyrate (C4) | +++ | +++ | + | + |
| Valeric (C5) | NG | NG | NG | NG |
| Decanoic (10) | + | NG | NG | NG |
| Dodecanoic (C12) (lauric) | +++ | + | + | NG |
| Myristic (C14) | NG | NG | NG | NG |
| Palmitic (C16) | NG | NG | NG | NG |
| Oleic (C18) | +++ | + | + | + |
| Glucose | ++++++ | ++++++ | ++++++ | ++++++ |

The following carbon sources were added to minimal medium with nitrate salts as the nitrogen source: acetate (50 mM); butyrate(10mM), propionate (0.1%), valerate (10 mM), decanoic, dodecanoic, palmitic and myristic (2.5mM), and glucose (1%) on agar plates and left at 24 C for 12 days.

NG (No growth), + very sparse growth, ++ sparse growth, +++ = poor growth, ++++ growth and conidiogenesis, +++++ good growth and conidiogenesis
